# The Synbiotic Combination of *Akkermansia muciniphila* and Quercetin Ameliorates Early Obesity and NAFLD through Gut Microbiota Reshaping and Bile Acid Metabolism Modulation

**DOI:** 10.3390/antiox10122001

**Published:** 2021-12-15

**Authors:** María Juárez-Fernández, David Porras, Petar Petrov, Sara Román-Sagüillo, María Victoria García-Mediavilla, Polina Soluyanova, Susana Martínez-Flórez, Javier González-Gallego, Esther Nistal, Ramiro Jover, Sonia Sánchez-Campos

**Affiliations:** 1 Institute of Biomedicine (IBIOMED), University of León, 24071 León, Spain; mjuarf@unileon.es (M.J.-F.); dpors@unileon.es (D.P.); sroms@unileon.es (S.R.-S.); mvgarm@unileon.es (M.V.G.-M.); s.martinez@unileon.es (S.M.-F.); jgonga@unileon.es (J.G.-G.); menisg@unileon.es (E.N.); 2 Experimental Hepatology Unit, IIS Hospital La Fe, 46026 Valencia, Spain; petar.petrov@ciberehd.org (P.P.); polina_soluyanova@iislafe.es (P.S.); ramiro.jover@uv.es (R.J.); 3 Centro de Investigación Biomédica en Red de Enfermedades Hepáticas y Digestivas (CIBERehd), Instituto de Salud Carlos III, 28029 Madrid, Spain; 4 Biochemistry and Molecular Biology Department, University of Valencia, 46010 Valencia, Spain

**Keywords:** *Akkermansia muciniphila*, childhood obesity, gut microbiota, quercetin, synbiotic

## Abstract

Gut microbiota plays a key role in obesity and non-alcoholic fatty liver disease (NAFLD), so synbiotics could be a therapeutic alternative. We aim to evaluate a nutritional intervention together with the administration of the bacteria *Akkermansia muciniphila* and the antioxidant quercetin in an in vivo model of early obesity and NAFLD. 21-day-old rats were fed with control or high-fat diet for six weeks. Then, all animals received control diet supplemented with/without quercetin and/or *A. muciniphila* for three weeks. Gut microbiota, NAFLD-related parameters, circulating bile acids (BAs) and liver gene expression were analyzed. The colonization with *A. muciniphila* was associated with less body fat, while synbiotic treatment caused a steatosis remission, linked to hepatic lipogenesis modulation. The synbiotic promoted higher abundance of *Cyanobacteria* and *Oscillospira*, and lower levels of *Actinobacteria*, *Lactococcus*, *Lactobacillus* and *Roseburia*. Moreover, it favored elevated unconjugated hydrophilic BAs plasma levels and enhanced hepatic expression of BA synthesis and transport genes. *A. muciniphila* correlated with circulating BAs and liver lipid and BA metabolism genes, suggesting a role of this bacterium in BA signaling. Beneficial effects of *A. muciniphila* and quercetin combination are driven by gut microbiota modulation, the shift in BAs and the gut-liver bile flow enhancement.

## 1. Introduction

Obesity is nowadays one of the major public health concerns worldwide and it is considered an international epidemic. The World Health Organization (WHO) estimates a 13% of prevalence in adults. This disease is continuously rising in children and adolescents, reaching numbers of 124 million worldwide in 2016 [1]. The pathogenesis is complex and multifactorial, being the sedentary patterns and the unhealthy habits the most important causes. Moreover, obesity is associated with many comorbidities, including metabolic syndrome and its hepatic manifestation, non-alcoholic fatty liver disease (NAFLD) [2].

NAFLD is one of the commonest manifestations of chronic hepatic disease and it affects around 20–30% of worldwide population. Nevertheless, this prevalence is much higher when obesity is considered, reaching numbers around 90% in adults and 40% in children [3]. According to the multiple-hit hypothesis [4], the pathogenesis of NAFLD is explained by the simultaneous action of many factors, including oxidative stress, insulin resistance or lipid metabolism alteration. In the last few years, gut microbiota has been identified as a key factor not only in NAFLD development, but also in obesity and type II diabetes [5,6,7].

Gut microbiota is considered as a metabolic organ that plays an essential role in human homeostasis and carries out many functions, among them the fermentation of carbohydrates and its transformation in short-chain fatty acids (SCFAs). Moreover, the gut microbiota makes an important contribution to the metabolism and the enterohepatic circulation of bile acids (BAs), transforming primary BAs into secondary metabolites, which represents a paradigmatic example of gut-liver axis communication [8]. Deconjugation by bile salt hydrolases followed by 7α-dehydroxylation, as well as the oxidation/epimerization by hydroxysteroid dehydrogenases are the major activities of intestinal bacteria on BAs [9]. The deconjugation and 7α-dehydroxylation increase the hydrophobicity of BAs and likely their toxicity [9]. Moreover, high concentrations of secondary BAs in feces, blood, and bile have been linked to several pathological conditions, including non-alcoholic steatohepatitis (NASH) [10].

Nowadays, due to the absence of an efficient therapy in obesity-associated NAFLD, the main strategy to its management is to change lifestyle towards healthy patterns, being nutritional interventions one of the first approaches [3]. Nevertheless, obesity-related NAFLD patients, especially children, usually have a low adherence to these type of interventions, and it is necessary to search for new strategies in the treatment of these pathologies [11,12]. Thus, due to the role of gut microbiota alterations in the development of NAFLD and obesity, the administration of prebiotics or probiotics are feasible therapies in the management of these diseases, and the combination of both, namely synbiotics, may potentiate the efficacy of the intervention [13,14]. In fact, the flavonoid quercetin has been reported to counteract NAFLD in in vivo models through a possible prebiotic effect, besides its antioxidant and anti-inflammatory properties [7,15]. Furthermore, the microorganism *Akkermansia muciniphila* has been described as a potential probiotic thanks to its protective effect in obesity development and its capacity to improve the gut barrier integrity [16,17,18]. In this sense, the safety role of pasteurized *A. muciniphila* as food ingredient has been recently confirmed [19] and approved by the European Food Safety Authority [20].

Therefore, quercetin and *A. muciniphila* combination in the form of synbiotic may have a synergetic effect in the management of obesity-related disorders. The aim of this study is to evaluate the combinatory effect of a nutritional intervention together with quercetin supplementation and *A. muciniphila* administration on gut microbiota composition, hepatic lipid and BA metabolism genes, circulating BAs, and NAFLD severity in an in vivo model of early obesity and NAFLD. 

## 2. Materials and Methods

### 2.1. Animals and Experimental Diets

**Figure 1:**
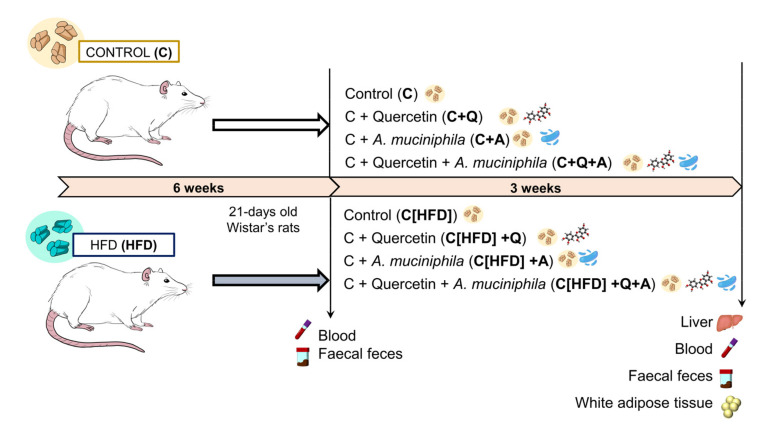
Experimental design. 21-day-old Wistar rats were fed with control or HFD for six weeks. Then, HFD-fed rats were allocated to control diet feeding and both groups (C and HFD) were split into four subgroups, based on diet intervention uniquely (C and Diet groups), quercetin supplementation (C+Q and C[HFD]+Q groups), *Akkermansia muciniphila* administration (C+A and C[HFD]+A groups) and combined quercetin supplementation and *A. muciniphila* administration (C+Q+A and C[HFD]+Q+A groups) for three weeks.

A total of sixty 21-day-old male Wistar rats were separated into two groups based on their diet (Research Diets, Inc. New Brunswick, NJ, USA): Control (C) (10% of energy from fat; D12450J) (*n* = 30) and semi-purified high fat diet (HFD) (60% energy from fat; D12492) (*n* = 30). After six weeks, HFD-fed rats were allocated to control diet feeding and both groups (C and HFD) were split into four subgroups, based on diet intervention uniquely (C [*n* = 7] and C[HFD] [*n* = 7] groups), quercetin supplementation (C+Q [*n* = 7] and C[HFD]+Q [*n* = 7] groups), *Akkermansia muciniphila* administration (C+A [*n* = 8] and C[HFD]+A [*n* = 8] groups) and combined quercetin supplementation and *A. muciniphila* administration (C+Q+A [*n* = 8] and C[HFD]+Q+A [*n* = 8] groups) (Figure 1). Rats were housed under controlled conditions of temperature, humidity, and lighting. The animals had free access to water and consumed the diet *ad libitum*. Body weight and food intake were weekly monitored. At the 9th week animals were euthanized by cardiac puncture under anesthesia. 

### 2.2. Dosage Information

Quercetin was supplemented in the diet (control and HFD diet) as aglycone quercetin (0.05 wt%) in a dosage of 37.5 mg kg^−1^ day^−1^ approximately. The human equivalent dose is roughly 6 mg kg^−1^ day^−1^, achievable through supplements or diets rich in fruits and vegetables. 

*Akkermansia muciniphila* was administrated by oral gavage at a dosage of 2 × 10^8^ CFU in 200 µL of cysteine supplemented (10%) skim milk as a vehicle. Non-*A. muciniphila* supplemented groups received daily administration of the vehicle alone.

### 2.3. Bacterial Strain and Growth Conditions

*Akkermansia muciniphila* (CIP-107961T) was cultured in a brain heart infusion medium (OXOID LTD, Bansingstoke, Hampshire, England) at 37 °C for 48 h under an anaerobic environment, as previously reported [21]. The cultures were centrifuged (9500 rpm for 10 min), washed twice with sterile phosphate buffer saline (PBS, pH 7.2), and then diluted to the final concentration of 2 × 10^8^ colony-forming units (CFU)/200 µL. The experiments described above were performed under strictly anaerobic conditions.

### 2.4. Sample Collection

Blood samples were taken for plasma isolation and subsequent biochemical analysis at 6th week and at the end of the experimental period. Liver and epididymal white adipose tissue were collected and weighted. Fecal and cecal content were collected for gut microbiota composition analysis and SCFAs quantitation. Portions of liver were immersed in preservative RNAlater for gene expression analysis. The right posterior lobe of the liver was fixed in 10% formalin for histological evaluation.

### 2.5. Histopathology

Paraffin-embedded liver samples were sectioned and stained with hematoxylin and eosin (H&E). The extent of hepatic lesions was evaluated according to the NAFLD activity score (*NAS*) to provide a numerical score based on three histological features: steatosis (0–3), lobular inflammation (0–3) and hepatocellular ballooning (0–2) [22]. The histological diagnostic was performed by two expert examiners blinded to experimental design protocol.

### 2.6. Biochemical Analysis

Alanine aminotransferase (ALT), aspartate aminotransferase (AST), and lactate dehydrogenase (LDH) activities, low-density lipoprotein (LDL), high-density lipoprotein (HDL) and total cholesterol levels were determined in plasma samples by the Instrumental Techniques Laboratory of the University of León using standard techniques. 

Insulin plasma concentration was determined using the commercial kit Rat/Mouse Insulin ELISA kit (EZRMI-13, Sigma-Aldrich; St. Luis, MO, USA), whereas leptin plasma concentration was quantified using Rat leptin ELISA kit (RAB0335, Sigma-Aldrich; St. Luis, MO, USA) according to manufacturer’s guidelines.

### 2.7. Fluorescent Microscopy

Frozen liver tissue samples were sectioned in 8–10 µm thickness sections using a cryostat. To analyse hepatic lipid accumulation, the sections were stained with 1 µg/mL Bodipy 493/503 (Invitrogen, Carlsbad, CA, USA) in combination with DAPI histology mounting medium for nuclei staining (Sigma-Aldrich, St. Louis, MO, USA). Sections were imaged by a Nikon Eclipse E600 Microscope with Y-FL EPI Fluorescence Attachment (Nikon, Tokio, Japan).

### 2.8. Hepatic Triglycerides Quantification

Frozen liver tissue (15−20 mg) was mixed with 450 μL of water in a 2 mL polypropylene tube for tissue homogenization using CK14 ceramic beads (Precellys, France) (two homogenization cycles of 25 s, 6000 rpm, 5 °C) in a Precellys 24 Dualsystem. Hepatic triglycerides isolation was carried out as previously described [23]. A colorimetric kit (Spinreact, Barcelona, Spain) was employed to analyze total hepatic triglyceride content. The concentration was expressed as μg/mg protein.

### 2.9. Gut Metagenomic Analysis

#### 2.9.1. Fecal DNA Extraction

The extraction of genomic DNA from fecal samples were carried out using Qiagen Fast DNA Stool Mini Kit (Qiagen, Hilden, Germany) according to manufacturer instructions, with some modifications, as previously described by us [7]. An initial bead-beating step was induced, and the lysis temperature was increased to 95 °C to aid in the recovery of DNA from bacteria that are difficult to lyse. DNA concentration was determined using a NanoDrop-1000 spectrophotometer (NanoDrop Technologies, Wilmington, DE, USA), and DNA samples were stored at −20 °C until further analysis.

#### 2.9.2. Amplification and Sequencing of 16S rRNA

Amplification of the 16S rRNA V3-V4 hypervariable region was performed by PCR as previously described [24], PCR assays were carried out in triplicate, and their products were pooled and purified with the Wizard^®^ Genomic DNA Purification Kit (Promega, Madison, WI, USA). Finally, amplicons were subjected to sequencing using the MiSeq reagent kit V3 in the Illumina MiSeq System according to the manufacturer’s instructions. 

#### 2.9.3. Bioinformatic Analysis

Illumina bcl2fastq© program was used to demultiplex sequencing data. Fastp and FastQC v0.11.8 (http://www.bioinformatics.babraham.ac.uk, accessed on 4 November 2021) tools allowed checking for quality, adapter trimmed and filtered forward and reversing raw reads.

QIIME software V1.9.1 MiSeq was used to analyze sequencing data [25], including forward and reverse reads joining, chimera removal, data filtering and taxonomic annotation. To remove chimeric sequences from the reads, the Usearch 6.1 algorithm was used [26]. Moreover, based on a 97% identity threshold value, reads were clustered into operational taxonomic units (OTUs). PyNAST was used for the alignment of the sequences with reference to the Greengenes core reference database (version 13_8) [27]. For taxonomic assignment, the UCLUST classifier was used [28]. The data were expressed as relative abundance.

### 2.10. Quantification of DNA and mRNA by Real-Time PCR

DNA obtained from fecal samples was amplified in a Step One Plus Real-Time PCR system (Applied Biosystems, Weiterstadt, Germany) using the SYBR Green I Master (Roche Diagnostics GmbH; Mannheim, Germany), and the primers listed in Table S1. Each sample was run in triplicate and total bacterial concentration determined by interpolation in a standard curve. Standards were created by amplifying the target 16S rRNA genes from appropriate positive control strains.

Isolation of total RNA from rat livers and epididymal white adipose tissue (WAT), cDNA synthesis, and quantitative real-time PCR (RT-qPCR) were performed essentially as described elsewhere [29]. A panel of 24 rat key mRNAs involved in hepatic BA and lipid metabolism, transport and regulation was selected for quantification [30]. Sequences of each primer pair are provided in Table S1.

Frozen rat liver samples (25–50 mg) were placed in 2-mL tubes containing CK14 ceramic beads (Precellys, Bertin Technologies, Montigny-le-Bretonneux, France) and 800 µL of RLT buffer (Qiagen, Hilden, Germany). Then, liver tissues were homogenized twice for 10 s at 6000 rpm at 4 °C in a Precellys 24 dual system equipped with a Criolys cooler. Tubes were centrifuged at 5600 rpm for 5 min at 4 °C. Next, total RNA was extracted from the supernatants with the RNeasy Plus Mini Kit (Qiagen, Hilden, Germany), according to manufacturer´s instructions. The final concentrations of RNA were determined by Nanodrop spectrophotometer (ND-1000, Thermo Fisher Scientific, Basingstoke, UK). 

Total RNA was purified from frozen WAT samples (100 mg) by using the RNeasy Lipid Tissue Mini Kit (Qiagen; Hilden, Germany) according to manufacturer´s instructions. 

For hepatic gene expression analyses, total RNA (1 µg) was reverse transcribed using the Moloney murine leukemia virus reverse transcriptase (Invitrogen, Waltham, MA, USA) and oligo-dT_14_ [31]. Diluted cDNA (2.5 ng cDNA equivalents per reaction) was amplified with a rapid thermal cycler (LightCycler Instrument LC480, Roche Applied Science, Mannheim, Germany) in 9.5 µL of 1× LightCycler DNA Master SYBR Green I (Roche Applied Science, Mannheim, Germany) and 0.5 µM of each primer. Data were normalized using the geometric mean of the mRNA concentration of the reference genes *Actb*, *Pgk1*, *Rplp0* and *Ptges3* as internal controls. Relative mRNA levels were calculated and expressed as fold-change over controls (value = 1.0), taking into account the efficiency of amplification for each gene [32]. The primers used in these analyses are listed in Table S1. 

For epididymal WAT gene expression analyses, total RNA (1 µg) was reverse transcribed, with a prior step of incubation with RQ1 RNase-free DNase (Promega, Madison, WI, USA) to remove residual genomic DNA. Diluted cDNA (1.25 ng cDNA equivalents per reaction) was amplified with the Step One Plus Real-Time PCR system (Applied Biosystems, Weiterstadt, Germany) in 10 µL of TaqMan Universal Master Mix II (Applied Biosystems; Waltham, MA, USA) and 1 µL of each probe (target gene and housekeeping) in a total volume per reaction of 20 µL. Glyceraldehyde-3-phosphate dehydrogenase (*Gapdh*) was used to normalize the cycle number at which the transcripts were detectable (Ct), referred to as ΔCt. TaqMan primers and probes were derived from the commercially available TaqMan^®^ Gene Expression Assays (Applied Biosystems) (*Gapdh*: 4352338E, *Plin2*: Rn01399516_m1, *Pparγ*: Rn00440945_m1)

### 2.11. Plasma Bile Acid Quantification

Bile acids (BAs) were profiled by an ultra-performance liquid chromatography/multiple reaction monitoring/mass spectrometry (UPLC-MRM-MS) method as described previously [33].

50 μL of rat plasma were spiked with deuterated internal standards. Then, proteins were precipitated, and supernatants were dried and reconstituted in 50 μL methanol:water (50:50, *v*/*v*). Samples were analyzed using an Acquity UPLC system (Waters, Wilmslow, UK) equipped with an Acquity UPLC BEH C18 column (1.7 μm, 2.1 × 100 mm; Waters). The MS analysis was performed using a Waters Xevo TQ-S mass spectrometer (Waters) with an ESI source working in the negative-ion mode. This method allows the quantification of 12 unconjugated, 8 glycine-conjugated, and 11 taurine-conjugated BAs, using 5 additional deuterated BAs as internal standards in a single analytical run. These analyses were performed in the Analytical Unit, Core Facility, IIS Hospital La Fe in Valencia, Spain.

### 2.12. Short-Chain Fatty Acids (SCFAs) Identification and Quantification

Identification and quantification of SCFAs were carried out using gas chromatography with flame ionization detection (GC-FID) technique. Approximately, 60 mg of faeces were diluted in 300 μL HCl (0.1%) and were stored at 0 °C for 25 min. After that, the mix was homogenized with vortex and was centrifugated at 15,000 rpm for 30 min. Samples were subsequently filtered with 0.2 µm nylon filter and analyzed by GC-FID.

### 2.13. Statistical Analysis

Data are expressed as the mean ± SEM. Significant differences were evaluated by Student’s *t* test (6th week) or multiple pairwise comparisons. *p* < 0.05 was considered to be significant for a difference. From gut microbiota composition, data statistical significance was determined by nonparametric Kruskal–Wallis test followed by Mann–Whitney U-test when *p* < 0.05. Permutational multivariate analysis of variance (PERMANOVA) was used to determine the significance of the different factors’ effects on bacterial communities’ distribution. Statistical analyzes were performed using SPSS 25.0 software (Chicago, IL, USA) and R software (R-project, Vienna, Austria). The Pearson’s correlation coefficient was used to examine correlations between gut microbiota composition, BA pool and liver gene expression.

### 2.14. Ethical Statement

All procedures were performed in accordance with the European Research Council guidelines for animal care and use and under the approval by the local Animal Ethics Committees.

## 3. Results

### 3.1. HFD Induces Juvenile Obesity and Metabolic Alterations in Rats

Six weeks of HFD feeding in juvenile rats resulted in significantly higher body weight than control diet-fed counterparts (+8.6%) compatible with juvenile obesity development (Table 1). The food intake of HFD group was significantly higher compared with C group, expressed as kcal per day (Table 1).

The biochemical analysis at the 6th week revealed a significant increase of ALT levels (+77.1%), LDL and total cholesterol concentration (+37.6% and +10.9%, respectively) in HFD-fed rats compared to the control group. Moreover, whereas fasting blood glucose was slightly but significantly decreased with HFD (−7.8%), insulin concentration was higher (+56.3%), which drifted into an insulin resistance, as it showed the increase of HOMA-IR index (+72.6%). The rest of the analysed parameters remained unchanged (Table 1).

### 3.2. Gut Microbiota Is Altered by HFD in Juvenile Rats

**Figure 2:**
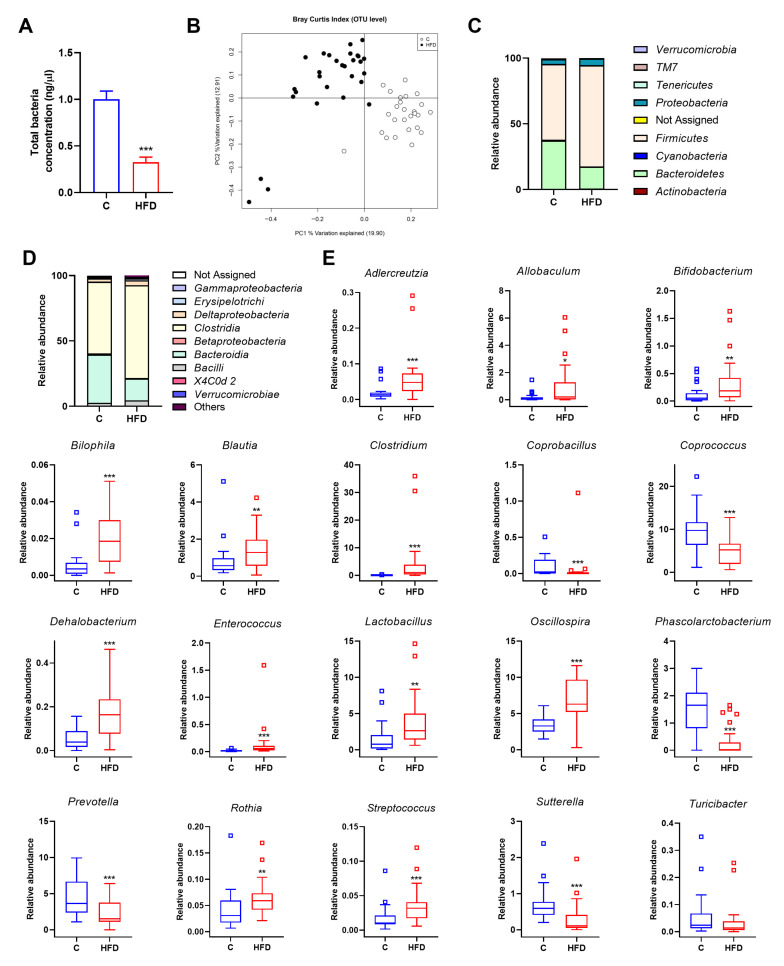
Effect of diet on microbiota composition in juvenile rats fed with control or HFD over six weeks. (**A**) Total bacteria concentration analysed by qPCR. Data represent mean ± SEM. *** *p* < 0.001 vs. C group. (**B**) Principal coordinates analysis (PCoA) plot based on Bray–Curtis dissimilarity index at the OTU level between rats fed with control or HFD. Percentage of the total variance explained is indicated in parenthesis in each axis. (**C**) Relative abundance at phylum level in the different experimental groups. (**D**) Relative abundance at class level in the different experimental groups. (**E**) Differences in the relative abundance between rats fed with control or HFD at genus level. Statistical analysis was performed using Kruskal–Wallis followed by Mann–Whitney U test (*p* < 0.05). * *p* < 0.05, ** *p* < 0.01, *** *p* < 0.001 vs. C.

HFD intake triggered a lower concentration of the total bacterial DNA in HFD-fed rats in comparison to control groups (Figure 2A), but increased gut microbiota diversity (Figure S1A). HFD also disturbed fecal production of SCFAs, showing a significantly higher valeric and isovaleric acids concentration in faeces in comparison to control group (Figure S1B). Principal coordinates analysis (PCoA) based on the Bray–Curtis index was performed to analyse the influence of diet at OTU level. The bacterial communities of HFD-fed rats clustered separately from the bacterial communities of control group according to the first axis, which accounted for 19.9% of the total variance (Figure 2B).

After six weeks, HFD significantly modified the microbiota profile of juvenile rats at phylum level. The relative abundance of *Firmicutes*, *Actinobacteria* and *Proteobacteria* were higher in HFD-fed rats in comparison to the control group, whereas *Cyanobacteria* and *Bacteroidetes* showed an opposite pattern (Figure 2C). Furthermore, the ratio *Firmicutes*/*Bacteroidetes* was significantly different between control and HFD groups.

Changes were also detected analysing microbiota composition at class level, being *Actinobacteria*, *Bacilli*, *Clostridia*, *Coriobacteria, Deltaproteobacteria* and *Erysipelothrix* significantly increased in HFD-fed rats. On the contrary, *Alphaproteobacteria*, *Bacteroidia* and *Betaproteobacteria* were considerably reduced in HFD group in comparison to control group (Figure 2D and Figure S1C).

As shown in Figure 2E, HFD also increased the relative abundance of *Adlercreutzia*, *Allobaculum*, *Bifidobacterium*, *Bilophila*, *Blautia*, *Clostridium*, *Dehalobacterium*, *Enterococcus*, *Lactobacillus*, *Oscillospira*, *Rothia* and *Streptococcus* genera and reduced the detection of *Coprobacillus*, *Coprococcus*, *Phascolarctobacterium*, *Prevotella*, *Roseburia*, *Sutterella* and *Turicibacter* genera, compared with control group (Figure 2E).

### 3.3. Quercetin and A. muciniphila Counteract HFD-Induced Obesity-Associated Features in Juvenile Rats

Quercetin and *A. muciniphila* supplementation in control groups did not modify any of the analyzed biochemical or physiological parameters, so these individuals were assimilated to a unique control diet group (C) for further comparisons.

**Figure 3:**
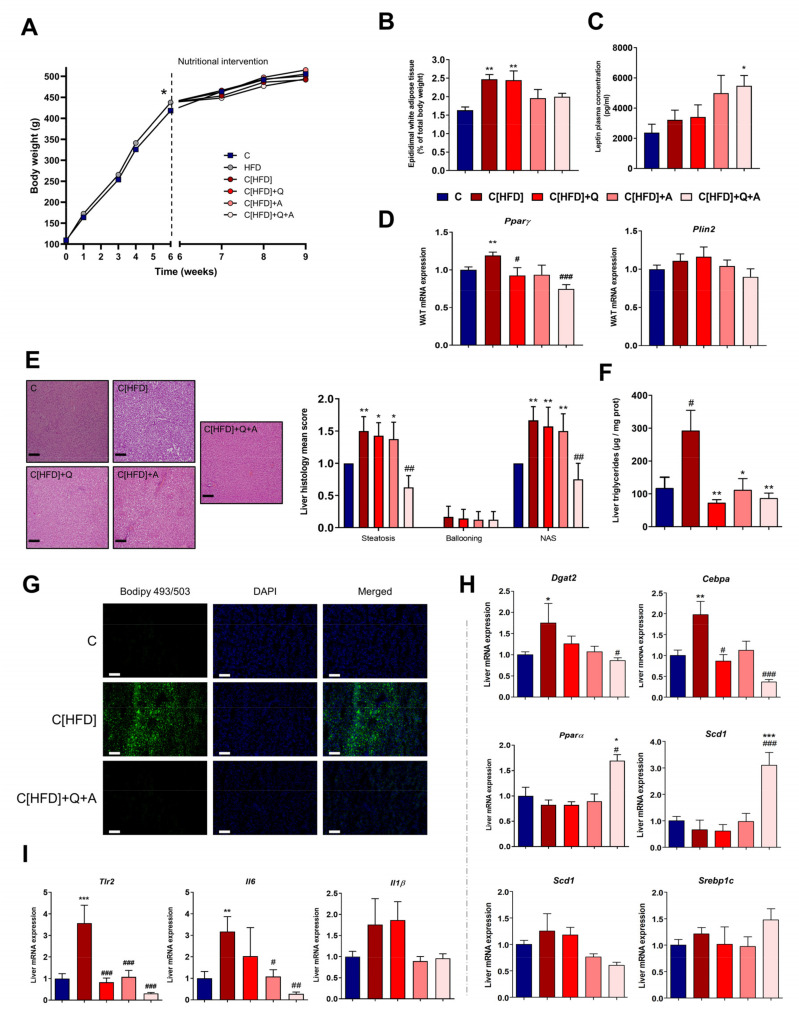
Effect of diet, quercetin and *A. muciniphila* supplementation on obesity and NAFLD-associated histological findings and hepatic lipid metabolism. (**A**) Body weight gain throughout the intervention. * *p* < 0.05 vs. C group at 6th week. (**B**) Epididymal white adipose tissue content. (**C**) Leptin plasma concentration (pg/mL). (**D**) Relative mRNA expression in epidydimal white adipose tissue of genes involved in lipid accumulation (*Pparγ* and *Plin2*). (**E**) Representative haematoxylin and eosin stained liver sections (scale bar: 100 µm) and NAFLD activity score (NAS) (calculated from individual scores for steatosis, lobular inflammation and ballooning). (**F**) Hepatic triglycerides quantification (μg/mg protein). (**G**) Representative images of neutral lipid staining with Bodipy 493/503 (green). Nuclei were stained with DAPI (blue) and merged images of both labelling are shown. Scale bar: 100 µm. (**H**) Relative mRNA expression in liver tissue of genes involved in inflammation process [proinflammatory cytokines (*Tlr2, Il6, Il1β*)]. (**I**) Relative mRNA expression in liver tissue of genes involved in lipid metabolism [transcription factors (*Cebpa*, *Pparα, Srebp1c*, *Srebp2*), lipid synthesis (*Dgat2* and *Scd1*)]. Bars represent mean ± SEM. * *p* < 0.05, ** *p* < 0.01, *** *p* < 0.001 vs. C; ^#^ *p* < 0.05, ^##^ *p* < 0.01, ^###^ *p* < 0.001 vs. C[HFD]. Statistical analysis was performed by one-way ANOVA followed by Tukey’s multiple comparison test.

No differences were found in total body weight gain at the end of the study between C and former HFD-fed rats (dietary intervention groups). However, the later groups tended to gain less body weight through the three weeks of dietary intervention, compensating the previously existing gap between groups (Figure 3A). The dietary intervention also counteracted the increase of plasmatic ALT activity, and total cholesterol levels observed after six weeks under HFD, as no significant differences in those parameters were found between groups at the end of the study (Table S2). However, fasting blood glucose and plasma insulin levels remained significantly increased in those rats under only dietary intervention (C[HFD]). In contrast, the combination of quercetin and *A. muciniphila* reduced the concentration of these parameters. Consequently, the HOMA-IR index, which was significantly higher in the C[HFD] group, significantly decreased after the synbiotic administration, pointing out altogether the potential of this intervention of reducing insulin resistance (Table S2). 

Moreover, the percentage of epididymal white adipose tissue (WAT) was higher in dietary intervention groups supplemented or not with quercetin alone (C[HFD] and C[HFD]+Q) with respect to control group, but not in both groups supplemented with *A. muciniphila* (C[HFD]+A and C[HFD]+Q+A, Figure 3B). Leptin concentration in plasma was measured after three weeks of dietary intervention, showing a significative increase in C[HFD]+Q+A group in comparison to C[HFD] (Figure 3C). Regarding the expression of key genes involved in lipid accumulation in white adipose tissue, the mRNA level of Peroxisome proliferator-activated receptor gamma (*Pparγ*), a primordial promotor of lipid uptake and adipogenesis, decreased significantly in C[HFD]+Q+A rats after three weeks of the synbiotic administration, while expression remained increased in C[HFD] despite the diet intervention (Figure 3D). The expression of Periplin 2 (*Plin2*), which is target of *Pparγ*, showed the same trend (Figure 3D). 

Despite dietary intervention, former HFD-fed groups still showed histological alterations associated to NAFLD development (steatosis and incipient ballooning), resulting in a higher NAS score, with the exception of the rats supplemented with both quercetin and *A. muciniphila* (Figure 3E). The rats subjected to the synbiotic treatment showed significantly less hepatic steatosis than the rest of the intervention groups, obtaining a NAS score similar to controls. Moreover, quercetin administration and *A. muciniphila* supplementation, and their combination, significantly reduced liver triglycerides concentration (Figure 3F). Related to that, staining of hepatic neutral lipids revealed a notable reduction in the number and size of lipid droplets in the group under the synbiotic, in comparison to the remarkable fat deposition observed in the rats subjected to dietary intervention alone (C[HFD], Figure 3G).

Gene expression analysis by RT-qPCR was conducted to determine if the hepatic lowering effect of the synbiotic intervention could be linked to lipid metabolism modulation. Transcription factor CCAAT/enhancer-binding protein alpha (*Cebpa*) level, which promotes adipogenesis, was overexpressed in the liver of C[HFD] rats, whereas complementation with either quercetin, *A. muciniphila* or both resulted in levels similar to the control group or even lower (Figure 3H). The final step of triacylglycerol synthesis is catalyzed by diacylglycerol O-acyltransferase (*Dgat*) 2, a target gene of C/EBPs. *Dgat2* profile expression in rat liver was very similar to *Cebpa*, as supplementation with the synbiotic repressed this enzyme, which agrees with the lack of hepatosteatosis in C[HFD]+Q+A rats (Figure 3H). In this sense, Peroxisome proliferator-activated receptor alpha (*Pparα*), which plays a crucial role in the regulation of fatty acid uptake and oxidation, lipid metabolism and insulin sensitivity, manifested a significant increase after 3-week administration of the synbiotic in comparison to C and C[HFD] groups (Figure 3H).

The expression profile of sterol regulatory element-binding protein (*Srebp*) 2, a regulator of cholesterol biosynthesis, was similar to *Cebpa,* but differences were not significant (Figure 3H). Contrarily, *Srebp1c* expression, a regulator of fatty acid synthesis, showed a trend to increase when the diet was supplemented with the synbiotic. In agreement, one of its target genes, Stearoyl-CoA desaturase-1 (*Scd1*), was also induced in these conditions (Figure 3H). 

Furthermore, to determine the effect of the synbiotic administration on the inflammatory status of the liver, RT-qPCR analysis was performed. C[HFD] liver mRNA expression of the proinflammatory markers Interleukin 6 (*Il6*) and Toll-Like Receptor 2 (*Tlr2*), remained significantly increased in comparison to C group after three weeks of dietary intervention, while a remarkable decrease in the expression of these genes was observed in the group under synbiotic supplementation (Figure 3I). The proinflammatory cytokine Interleukin 1β (*Il1β*) showed a similar pattern, pointing out a reduction in the hepatic inflammatory status in the animals subjected to dietary intervention and synbiotic administration. 

### 3.4. Effect of Diet, Quercetin and A. muciniphila Supplementation on Gut Microbiota Composition in the Early Obesity and NAFLD Model

**Figure 4:**
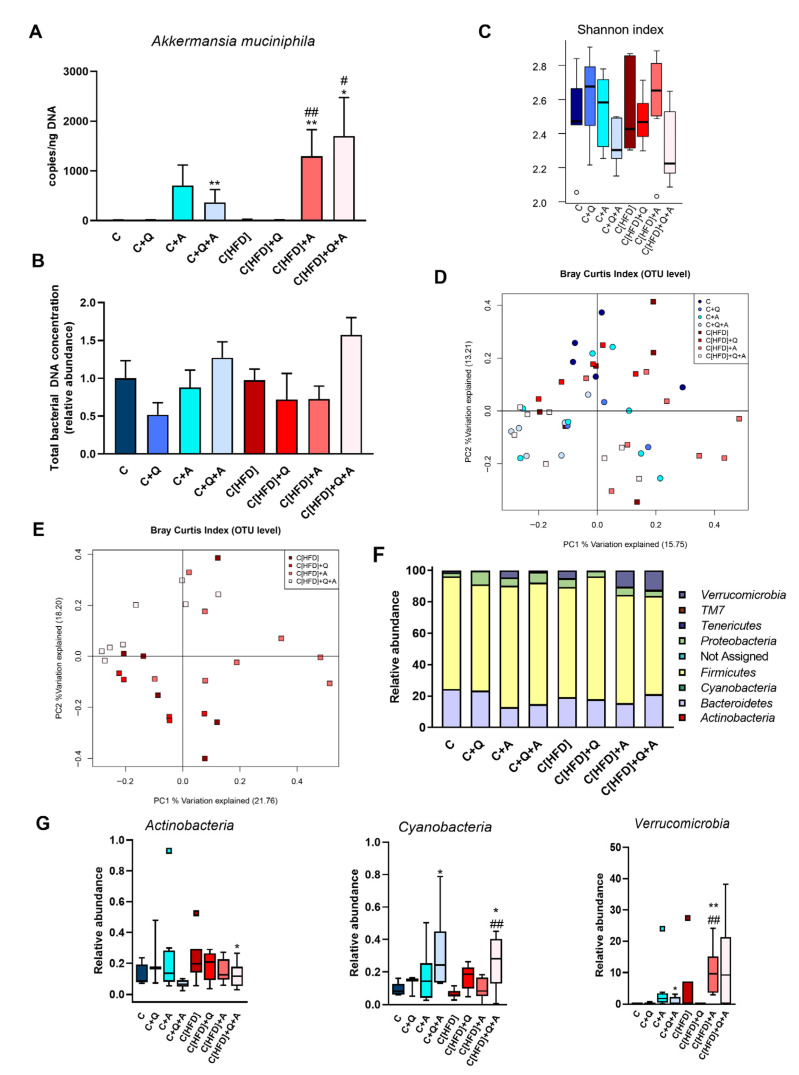
Metagenomic findings in rats after three weeks of dietary intervention, quercetin and *A. muciniphila* supplementation. (**A**) *Akkermansia muciniphila* quantification by qPCR. (**B**) Total bacterial concentration in fecal samples determined by qPCR (relative amount). (**C**) α-diversity measured by Shannon index at the end of the study. (**D**) Principal Coordinates Analysis (PCoA) plot based on Bray–Curtis dissimilarity index at OTU level considering all experimental groups. Percentage of the total variance explained is indicated in parenthesis in each axis. (**E**) Principal Coordinates Analysis (PCoA) plot based on Bray–Curtis dissimilarity index at OTU level in HFD fed rats subjected to dietary intervention, quercetin and *A. muciniphila* supplementation. Percentage of the total variance explained is indicated in parenthesis. (**F**) Relative abundance of the total microbial population at phylum level. (**G**) Differences in the relative abundance of *Actinobacteria*, *Cyanobacteria* and *Verrucomicrobia* phylum between C and HFD groups at 9th week. Statistical analysis was performed using Kruskal–Wallis followed by Mann–Whitney U-test (*p* < 0.05). * *p* < 0.05, ** *p* < 0.01 vs. C; ^#^ *p* < 0.05, ^##^ *p* < 0.01 vs. C[HFD].

*A. muciniphila* supplementation increased the copy number of this microorganism in the experimental groups subjected to the colonization. However, comparing the groups initially on control diet and HFD, the colonization seemed higher in HFD-fed groups (Figure 4A).

No significant differences in the total bacterial concentration and α-diversity were found between all the experimental groups at the end of the study, although the group supplemented with the synbiotic showed the highest total bacteria counts and a slightly decreased Shannon index (Figure 4B,C). The fecal acetate, propionate and butyrate levels significantly increased in former HFD-fed groups after 3-weeks of intervention, but no differences between groups were found at the end of the study (Figure S2).

A principal coordinate analysis (PCoA) based on the Bray Curtis index was performed to analyze the individual influence of the diet, quercetin and *A. muciniphila* supplementation or their combination on the distribution of bacterial communities at OTU level. The diet-specific pattern identified at 6th week was not observed at the end of the study, suggesting that three weeks of nutritional intervention was sufficient to homogenize bacterial communities (Figure 4D). To further determine the effect of quercetin and *A. muciniphila* administration on bacterial communities of formerly HFD-fed groups, an additional PCoA considering only those groups was conducted. Although some data points were dispersed along the graph, the subjects receiving the synbiotic tended to cluster together and separated from the other groups in the upper left quadrant (Figure 4E). Statistical analysis supported that both *A. muciniphila* (F = 2.5383, *p* = 0.0064) and quercetin supplementation (F = 2.3317, *p* = 0.0141) contributed significantly to the observed distribution of bacterial communities.

**Figure 5:**
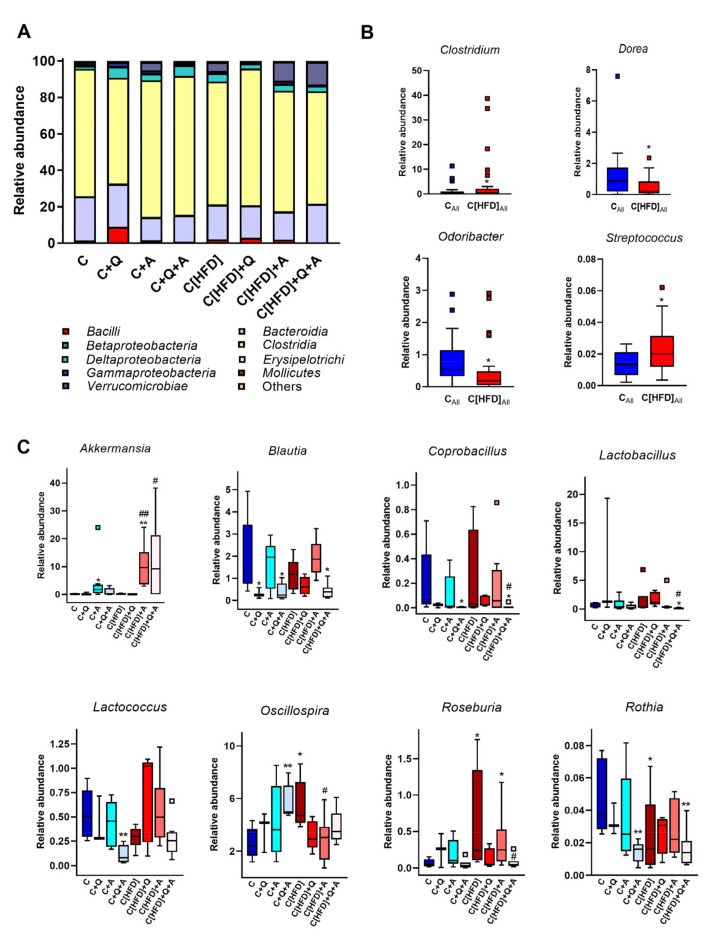
Metagenomic findings in rats after three weeks of dietary intervention, quercetin and *A. muciniphila* supplementation. (**A**) Relative abundance of the total microbial population at class level. (**B**) Differences in the relative abundance at genus level between control diet-fed or formerly HFD-fed rats independently of other treatments. * *p* < 0.05 vs. control diet-fed group (C_All_). (**C**) Differences in the relative abundance at genus level considering all experimental groups. Statistical analysis was performed using Kruskal–Wallis followed by Mann–Whitney U-test (*p* < 0.05). * *p* < 0.05, ** *p* < 0.01, vs. C; ^#^ *p* < 0.05, ^##^ *p*< 0.01 vs. C[HFD].

Metagenomic analysis revealed significant differences at phylum, class, and genus levels after three weeks of the experiment. No changes associated to the initial 6-week HFD feeding were found at the phylum level. *A. muciniphila* intervention was associated with a notable increase in the abundance of *Verrucomicrobia* phylum in all the supplemented groups, corroborating the results obtained by direct quantification of this microorganism. Moreover, the combination of *A. muciniphila* and quercetin administration was associated with a significant increase in the relative abundance of *Cyanobacteria* phylum, as well as a decrease in *Actinobacteria* phylum (Figure 4F,G). Similar results were obtained at class level, showing a significantly higher detection of *Verrucomicrobiae* after *A. muciniphila* supplementation. Additionally, *Bacilli* class, which was increased in HFD fed rats, was reduced with the combination of quercetin and *A. muciniphila* (Figure 5A).

Despite the nutritional intervention with control diet performed for three weeks, some differences at genus level were observed associated with the previous 6-week HFD. Thus, a higher abundance of *Streptococcus* and *Clostridium* genera was detected, as well as a decrease of *Odoribacter* and *Dorea* genera (Figure 5B). As expected, *A. muciniphila* supplementation was associated with a higher abundance of *Akkermansia* genus (Figure 5C). Additionally, *Blautia* and *Coprobacillus* genera were reduced with quercetin supplementation, both in single supplementation or in combination with *A. muciniphila* and independently of the previous type of feeding. Furthermore, several differences at genus level were observed in the synbiotic supplemented groups that were not detected with the administration of any of the treatments alone. Thus, the synbiotic reduced the detection of *Lactobacillus*, *Lactococcus* and *Rothia* genera, whereas *Oscillospira* was highly abundant in both supplemented groups. Finally, the abundance of *Roseburia*, increased in the C[HFD] group, was significantly reduced in the rats subjected to *A. muciniphila* and quercetin combination (Figure 5C).

### 3.5. Effects of the Interventions on Plasma Bile Acid Levels

**Figure 6:**
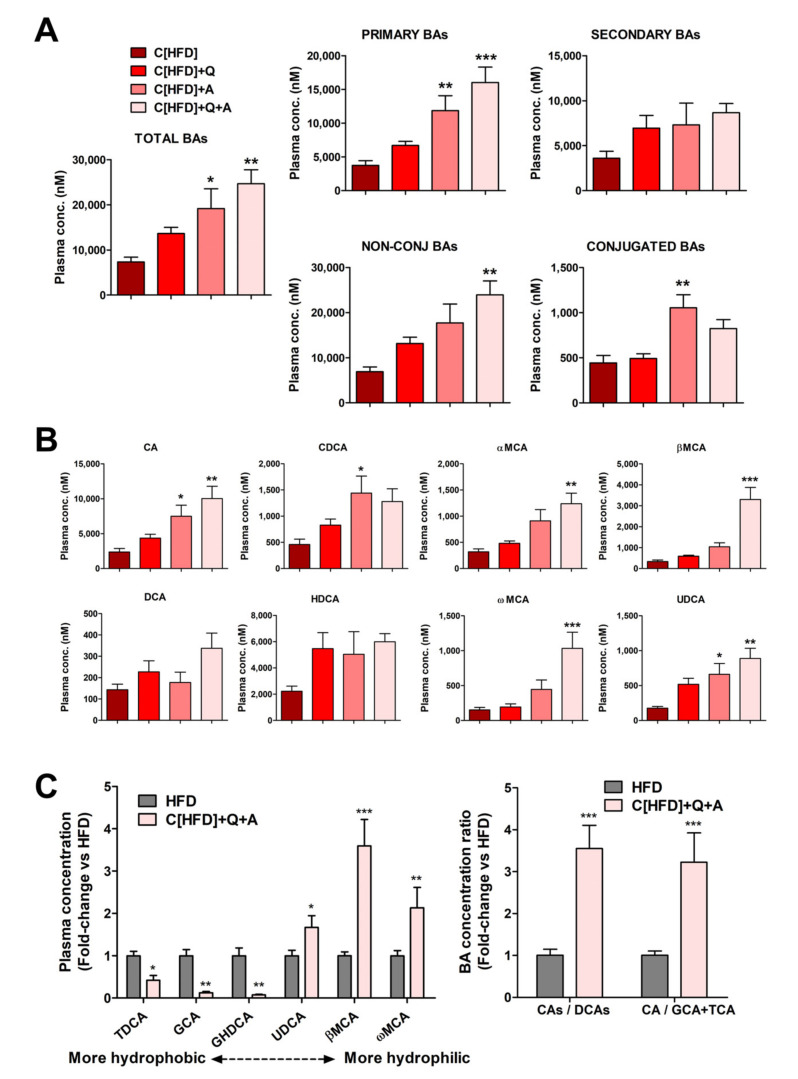
Effect of diet, quercetin and *A. muciniphila* supplementation on plasma BA levels in a juvenile rat NAFLD model. (**A**) Concentrations (nM) of total, primary, secondary, conjugated and unconjugated BAs. (**B**) Specific primary (CA, CDCA and muricholic acids αMCA and βMCA) and secondary (DCA, HDCA, ωMCA and UDCA) BA concentrations. (**C**, **left**) Relative plasma concentration of hydrophobic and hydrophilic Bas in HFD group and in HFD group after control diet supplemented with quercetin and *A. muciniphila*. (**C**, **right**) BA concentration ratios of primary CA+TCA+GCA vs. secondary derivatives DCA+TDCA+GDCA, and CA vs. conjugated CA species (GCA+TCA), in HFD group and in HFD group after control diet supplemented with quercetin and *A. muciniphila*. Data represent mean of absolute values ± SEM (**A**,**B**), or mean of normalized to HFD values ± SEM (**C**). * *p* < 0.05, ** *p* < 0.01, *** *p* < 0.001, vs. C[HFD] (**A**,**B**) or vs. HFD group (**C**). Statistical analysis was performed by one-way ANOVA followed by Tukey’s multiple comparison test.

Quantification of plasma BAs demonstrated that diet supplementation with quercetin and *A. muciniphila* (C[HFD]+Q+A) triggered a significant additive increase in total plasma BA levels (Figure 6A), primarily due to a higher concentration of primary BAs. A significant alteration in total secondary BAs was not observed (Figure 6A), which likely indicates that diet supplementation with the synbiotic favors primary BAs synthesis in the liver. 

Changes in BA conjugation with glycine and taurine were also analyzed. Diet supplementation with quercetin and *A. muciniphila* triggered a more important increase in unconjugated than in conjugated BAs (Figure 6A), and their ratio raised (NC/C: 17.7 in C[HFD] vs. 30.6 in C[HFD]+Q+A). 

Regarding specific BAs, all unconjugated primary species were increased in plasma after the combined quercetin and *A. muciniphila* administration, with cholic acid (CA), β-muricholic acid (βMCA) and α-muricholic acid (αMCA) being the most induced (Figure 6B). Regarding unconjugated secondary BAs, the more hydrophobic (deoxycholic acid (DCA) and hyodeoxycholic acid (HDCA)) did not significantly change, whereas the more hydrophilic (ω-muricholic acid (ωMCA) and ursodeoxycholic acid (UDCA)) were also induced upon the synbiotic (Figure 6B).

Plasma BAs levels before and after the intervention were also investigated. The total BA concentration was 2.76-fold higher in the 6-week HFD rats (HFD) than in the 6-week HFD + 3-week control diet rats (C[HFD]) (20,243 ± 6124 nM vs. 7332 ± 2850 nM, *p* < 0.001). The most remarkable differences were observed in conjugated (i.e., GCA, GDCA and GHDCA) and in some secondary (i.e., DCA) BAs, all of them being much more elevated in HFD rats before intervention (data not shown). The BA profiles before and after synbiotic administration were also compared. The intervention with control diet supplemented with the synbiotic resulted in reduced levels of hydrophobic BAs (TDCA, GCA and GHDCA) along with increased levels of more hydrophilic ones (UDCA, βMCA and ωMCA) (Figure 6C). The ratio between primary cholic acid+salts (CA+GCA+TCA) and their secondary deoxycholic acid + salts (DCA+GDCA+TDCA) increased more than 3-fold. Similarly, the ratio between CA and its conjugated salts (GCA+TCA) also increased more than 3-fold (Figure 6C). The increases in these ratios point again to less hydrophobic unconjugated plasma BAs after quercetin and *A. muciniphila* combination.

### 3.6. Effects of the Interventions on the Hepatic Expression of Bile Acid Synthesis, Metabolism and Transport Related Genes

Quercetin and *A. muciniphila* supplementation after six weeks of control diet did not significantly modify the expression of the 22 rat liver mRNAs quantified; therefore, the results from these animals were averaged in a unique control diet group (C) for further comparisons.

**Figure 7:**
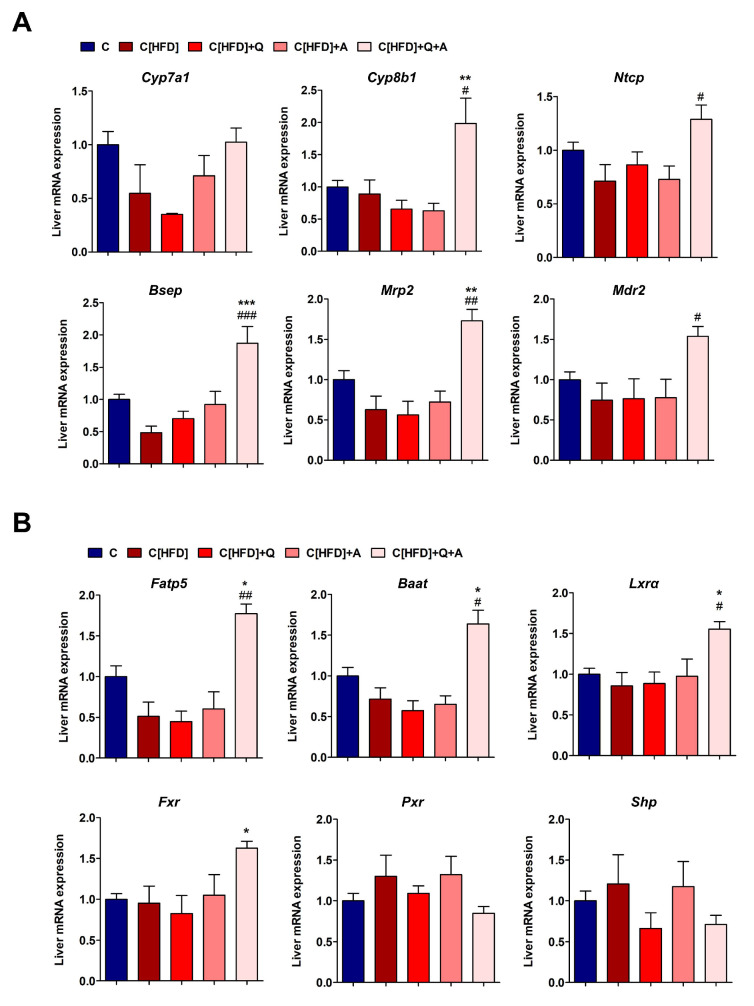
Effect of diet, quercetin and *A. muciniphila* supplementation on the expression of BA and lipid metabolism genes in the liver of juvenile NAFLD rats. (**A**) Liver mRNA expression of main BA synthesis genes (*Cyp7a1* and *Cyp8b1*), and of major basolateral BA uptake (*Ntcp*) and canalicular bile efflux (*Bsep*, *Mrp2* and *Mdr2*) transporters. (**B**) Expression of the BA-CoA ligase (*Fatp5*), the BA-CoA:amino acid N-acyltransferase (*Baat*), and of the BA-related nuclear receptors *Fxr*, *Pxr*, *Lxrα*, and *Shp*. Expression of mRNA is normalized to the mean values of C rats. Data represent mean ± SEM. * *p* < 0.05, ** *p* < 0.01, *** *p* < 0.001, vs. C; ^#^ *p* < 0.05, ^##^ *p* < 0.01, ^###^ *p* < 0.001, vs. C[HFD] by one-way ANOVA followed by Tukey’s multiple comparison test.

Contrarily, diet supplementation with quercetin and *A. muciniphila* after six weeks of HFD had significant effects on the expression of many of these mRNAs. Regarding BA synthesis, the synbiotic approach upregulated *Cyp7a1* and *Cyp8b1* expression, yet the increase in *Cyp7a1* did not reach statistical significance (Figure 7A). Regarding hepatocyte BA transporters, key basolateral *Ntcp* (BA uptake) and canalicular *Bsep* and *Mrp2* (BA efflux) transporters were significantly induced after the synbiotic intervention. Another important component for proper bile formation, the canalicular phospholipid flippase *Mdr2*, was also induced by the combined treatment. These results suggest increased hepatocellular BA synthesis and transport in rats subjected to quercetin and *A. muciniphila* intervention upon HFD (Figure 7A).

The BA-CoA synthetase *Fatp5* and the BA-CoA:amino acid N-acyltransferase *Baat* mRNAs were both upregulated in C[HFD]+Q+A rat livers, which is indicative of an increased BA conjugation activity (Figure 7B). This could be an adaptive response to deal with the higher level of plasma unconjugated BAs observed in these animals.

The expression of several nuclear receptors involved in BA homeostasis was also investigated. The mRNA levels of *Lxrα* and *Fxr* were significantly upregulated in C[HFD]+Q+A rat livers at the end of the study (Figure 7B). However, the bona fide *Fxr* target gene, *Shp*, did not show a similar profile. Both *Shp* and *Pxr* did not vary significantly, yet they had a trend to decrease in C[HFD]+Q+A rats (Figure 7B).

### 3.7. Associations among Gut Microbiota, Plasma Bile Acids and Liver Gene Expression

**Figure 8:**
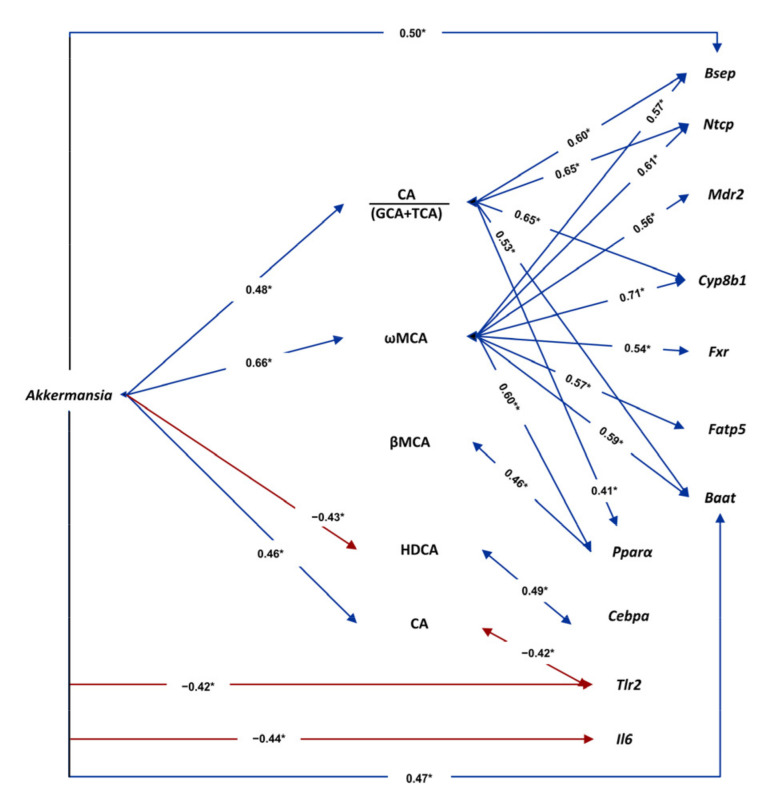
Associations of gut *Akkermansia* genus with plasma BAs and with hepatic gene expression. The values in bidirectional arrows represent the Pearson’s correlation coefficients; * *p* < 0.05. Detailed XY representation of each association is provided in the Figure S3.

Pearson correlation analyses showed potential associations between gut microbiota, plasma BA levels and liver gene expression. The genus *Akkermansia* (and its class and phylum) showed numerous significant correlations. Among them, a positive association with CA and with the ratio CA/GCA+TCA (suggesting influence in BA deconjugation); another positive association with the more hydrophilic ωMCA; and a negative correlation with the more hydrophobic HDCA (Figure 8 and Figure S3). Regarding liver gene expression, a negative correlation of *Akkermansia* with the proinflammatory mediators *Tlr2* and *Il6* was observed (Figure 8 and Figure S3). ωMCA, in turn, showed a significant positive correlation with liver genes involved in bile flow (*Ntcp*, *Bsep* and *Mdr2*), BA synthesis (*Cyp8b1*), BA conjugation (*Fatp5* and *Baat*) and BA regulation (*Fxr*), as well as with the liver gene involved in the regulation of fatty acid uptake and oxidation *(Pparα*). On the contrary, HDCA is positively associated with the lipogenic factor *Cebpa* (Figure 8 and Figure S3). Furthermore, CA was negatively associated with *Tlr2*, whereas the ratio CA/GCA+TCA was positively associated with *Pparα* (Figure 8 and Figure S3).

*Cyanobacteria* phylum (induced in C[HFD]+Q+A rats) significantly correlated with the CA/DCA ratio (r = 0.44, suggesting negative effect on DCA synthesis), whereas genus *Coprobacillus* (repressed in C[HFD]+Q+A rats) showed the opposite trend: a significant correlation with DCA (r = 0.64). Moreover, *Coprobacillus* genus also showed a significant correlation with the hepatic expression of *Pparα* (r = −0.66) and *Il6* (r = 0.66). *Roseburia* genus (repressed in C[HFD]+Q+A rats) showed a negative correlation with total primary BAs (r = −0.46) and the primary αMCA (r = −0.42), a positive correlation with the secondary HDCA (r = 0.46), and a positive correlation with the proinflammatory cytokine *Il6* (r = 0.48) (Table S3).

The hepatic expression of *Cebpa* (repressed in C[HFD]+Q+A rats) correlated positively with *Actinobacteria* phylum (r = 0.40), but negatively with *Cyanobacteria* phylum (r = −0.45), the ratio CA/DCA (r = −0.40) and the primary βMCA (r = −0.55). *Srebp2* (repressed in C[HFD]+Q+A rats) also correlated positively with *Actinobacteria* (r = 0.43) and negatively with βMCA (r = −0.42). Moreover, *Actinobacteria* phylum also correlated with the hepatic expression of the proinflammatory cytokine *Il1β* (r = 0.45) (Table S3).

## 4. Discussion

Obesity and NAFLD development is strongly associated with a profound modification of the gut microbiota in all stages of the life [5,34]. The rising prevalence of these pathologies, and the lack of proper clinical management of patients besides lifestyle guidelines, which has a significant probability of failing, draws attention to potential supplements that may help to improve the outcomes of the treatments [35,36]. In this study, we developed a model of juvenile obesity-related NAFLD to evaluate the repercussion of the administration of the flavonoid quercetin and *Akkermansia muciniphila* on gut microbiota composition, hepatic lipid and BA metabolism gene modulation, and BA flow.

HFD feeding for six weeks induced a significant increase of both body weight gain, in accordance to juvenile obesity development, and biochemical parameters such as ALT and LDL, suggesting altogether an induced metabolic alteration in concordance with our previous results [24]. Besides, HFD diet group presented a remarkable increase in plasma insulin concentration and HOMA-IR index, which is closely linked to hepatic insulin resistance in NAFLD [37]. Additionally, HFD feeding also induced a gut microbiota imbalance at all taxonomic levels. *Firmicutes*, *Actinobacteria* and *Proteobacteria* were increased in HFD-fed rats, whereas *Cyanobacteria* and *Bacteroidetes* decreased, showing an increased *Firmicutes/Bacteroidetes* ratio, as previously described [24,38]. Dysbiosis was also observed at genus level, displaying genera such as *Blautia* and *Lactobacillus* significant increases in HFD-fed rats. High levels of *Lactobacillus* genus has thoroughly been identified in obesity and NAFLD [5,38,39], while a rise in *Blautia* has been related with NAFLD in pediatric patients [40].

Dietary intervention for three weeks with a control diet promoted a significant improvement in body weight gain, biochemical parameters and gut dysbiosis. The administration of the synbiotic for three weeks significantly diminished HOMA-IR index, pointing out the beneficial effect of *A. muciniphila* and quercetin on insulin resistance, as it has been previously described [7,17]. The differences at phylum, class and genus levels observed in our in vivo model were partially reverted after dietary intervention. Nevertheless, *Streptococcus* and *Clostridium* genera, which were augmented with HFD, remained increased despite three weeks of control diet. In this sense, both genera have been related with NAFLD development in in vivo models [7,24,41] and patients [42,43]. In fact, our research group has previously identified an association between *Clostridium* genus and an altered BA fecal metabolome [24], whereas *Streptococcus* has been linked with NASH or even cirrhosis and hepatocarcinoma development [40,44]. 

Additional and important benefits were observed with the synbiotic. In this regard, *A. muciniphila* administration reduced white adipose tissue and increased plasma leptin concentration. Previous studies have revealed a reduction of body fat mass and an amelioration of adipose tissue inflammation under *A. muciniphila* administration, supporting our findings [16,45,46]. Despite the fact that high leptin plasmatic levels are linked somehow to obesity, the higher leptin concentration in plasma after three weeks of the synbiotic administration in the absence of other metabolic disorders such as insulin resistance, could be oriented to promoting energy expenditure and controlling food intake to compensate the metabolic imbalance caused by HFD diet [47]. Regarding white adipose tissue status, the relative expression of *Pparγ*, which is involved in adipogenesis and adipose tissue differentiation processes, and *Plin2*, implicated in lipid storage, after 3-week administration of the synbiotic was similar to the control group, while the dietary intervention group showed significantly higher levels. In this sense, decreased expression of two genes has been associated to a protective role against obesity, insulin resistance and NAFLD development [48,49]. Moreover, incipient steatosis was observed in the non-supplemented HFD-fed group at the end of the study, while synbiotic administration significantly diminished the hepatic fat content. 

Additionally, the HFD-induced upregulation of *Cebpa*, *Dgat2* and *Srebp2*, involved in *de novo* lipogenesis and NAFLD, was significantly counteracted by the synbiotic combination [30,37,50]. However, an opposite trend was observed for *Srebp*, *Scd1* and *Pparα* gene expression. In this sense, the upregulation of *Srebp1c*, could be explained by the lack of fatty acid negative feedback, in these rats without hepatosteatosis. The *Srebp1c* target gene *Scd1*, responsible for fatty acid desaturation, has been associated with NAFLD development [51], but its upregulation could also be beneficial as monounsaturated fatty acids prevent the negative effects of saturated fatty acids, such as apoptosis and NASH [52,53]. Moreover, *Pparα* modulates lipid metabolism, promotes fatty acid beta-oxidation and regulates lipid fatty acid circulation, [54,55], thus its upregulation could result in a reduction of available fatty acid for triglyceride synthesis and an interruption of NAFLD progression. 

Regarding the expression of proinflammatory markers, a protective role of the synbiotic administration on the inflammatory status was observed, confirming the effect of quercetin or *A. muciniphila* supplementation previously reported [7,17]. Overall, our results suggest a role of this potential synbiotic in the management of juvenile obesity and NAFLD through the modulation of hepatic lipid metabolism, hepatic inflammatory status and white adipose tissue modulation.

Beneficial effects of the synbiotic were also reflected in the gut microbiota composition. As expected, the abundance of *Verrucomicrobia* phylum and *Akkermansia* genus increased drastically in the experimental groups supplemented with cultured *A. muciniphila*, demonstrating the gut colonization of this microorganism upon oral administration. Interestingly, the colonization seemed to be more successful in previously HFD-fed rats suggesting that the microenvironment created due to dietary patterns determined the ability of *A. muciniphila* to proliferate within its microbiota environment. Furthermore, some genera abundance was significantly modified in all quercetin-supplemented experimental groups, highlighting the decrease of *Blautia,* a genus highly detected in NAFLD patients, not only in adults but in pediatric patients too [40,56], and *Coprobacillus,* whose relative abundance has been previously associated with HFD [57]. Moreover, the relative abundance of *Cyanobacteria* phylum, which was reduced in HFD-fed rats, was increased due to the synbiotic supplementation, whereas *Actinobacteria* phylum, whose rise has been pointed out as a common marker of obesity [58], decreased with this combination. At genus level, *Lactobacillus* and *Lactococcus* suffered a significant decrease with the supplementation of quercetin and *A. muciniphila*. Previous studies in animal models and in humans have shown that obesity associates with high levels of *Lactobacillus* and *Lactococcus* genera, which also positively correlated with fasting plasma insulin [56,59]. In addition, supplementation with the synbiotic increased *Oscillospira* genus relative abundance in both control and HFD groups, which negatively correlates with hepatic fat deposition and shows a marginal detection in NAFLD and obese patients [60,61]. Our results reinforce the beneficial capacity of the synbiotic treatment in obesity and NAFLD development by a potential mechanism involving gut microbiota reshaping.

BAs are one of the main gut microbiota metabolites with critical metabolic roles. Indeed, the alteration of BA homeostasis contributes to the development of metabolic diseases such as obesity and NAFLD [62,63]. The BA profile observed in NAFLD and obese patients shows different perturbations depending on the stage of the disease and the patients’ age [64,65]. Jiao et al. [10] showed that primary and secondary BAs were elevated in the serum of patients with NAFLD, with secondary DCA disproportionally increased. Moreover, Puri et al. [62] reported an increase in conjugated BAs and, thus, decreased ratios CA/GCA+TCA and CDCA/GCDCA+TCDCA. In agreement, Caussy et al. [66] found that total unconjugated BAs significantly decreased along with NASH and fibrosis progression. In summary, the research in NAFLD adult patients indicates that total BA levels are increased, mostly by conjugated and secondary BAs [67]. In our juvenile obesity and NAFLD model we observed that diet intervention along with *A. muciniphila* and quercetin triggered an increase in the total plasma BA pool, being primary and unconjugated hydrophilic BAs (particularly muricholic acids (MCAs)) the responsible species. We also observed a rise of the primary CA with the synbiotic approach, whose role in obesity and NAFLD is contradictory. An increased CA/CDCA ratio was associated with human obesity [63]. In contrast, dietary CA supplementation improved glucose tolerance, decreased triglyceride accumulation in the liver and reduced HFD-induced weight gain in mice [68]. Moreover, we have observed a positive correlation between *Akkermansia* genus and CA concentration, which had been previously reported [69], as well as a negative correlation between this bile acid and *Tlr2* expression, suggesting a possible protective effect on inflammatory status. Moreover, a role of *Lactobacillus* genus (reduced by the synbiotic) in the transformation of CA and CDCA into DCA and LCA has also been reported [70].

In our obesity related-NAFLD model, a significant and positive correlation between CA/(GCA+TCA) ratio and *Akkermansia* genus was also identified. This could be explained by enhanced GCA and TCA deconjugation by gut microbiota bile salt hydrolase (BSH) activity. *Bacteroides, Clostridium, Bifidobacterium* and *Lactobacillus* are the main genera with such activity, while no such activity has been described in *Akkermansia* [8,10]. Our intervention did not increase these genera and, therefore, the association of *Akkermansia* with the CA/GCA+TCA ratio likely depends on increased *de novo* synthesis of unconjugated CA. Nevertheless, more studies are needed as a possible BSH activity in *Akkermansia* cannot be ruled out. Additionally, a negative correlation between HDCA levels and *Akkermansia* genus was observed, as well as a positive correlation between this BA and the hepatic expression of *Cebpa,* which supports a potential role of BAs as mediators in gut-liver communication. 

Elevated levels of MCA species lead to a less toxic and hydrophobic BA pool, which has been shown to improve obesity, insulin resistance, and NAFLD [71,72]. In this sense, gut microbiota is a crucial factor influencing the synthesis of these BAs in the liver and the conversion of αMCA into βMCA [73,74], which was remarkably increased in our study. Moreover, ωMCA plasma concentration was also augmented with the synbiotic approach and positively correlated with *Akkermansia* genus, suggesting that the gut microbiota reshaped by the intervention could be involved in the conversion of βMCA into ωMCA. Thus, all the beneficial effects of MCAs on NAFLD could reinforce the use of the intervention in the management of this disease. 

The modification in the plasma BA profile triggered by the synbiotic approach agreed with adaptations of BA pathways in the liver. BA synthesis feedback inhibition is exerted by FXR-agonistic BAs. However, in rodents, a potent positive feedback mechanism mediated by FXR-antagonistic MCAs is at least as important as that of FXR-agonistic BAs [75], and could explain the upregulation of the BA synthesis genes and the BA uptake transporter NTCP [76], which are under negative feedback control by FXR-SHP. In agreement, SHP showed a reduced expression trend in C[HFD]+Q+A rats. The proposed inhibition of FXR by MCAs should also result in reduced intestinal fibroblast growth factor 19 (FGF19) synthesis and secretion. This important hormone-like regulator was not investigated in this study, yet others have shown that serum FGF19 was increased in patients with NASH-HCC and associated with tumor markers and with NAFLD progression [77].

The synbiotic may induce changes in gut microbiota, which in turn activate BA synthesis and transport in the liver, thus establishing a healthier hydrophilic BA pool. Correlation analysis suggested that *Akkermansia* genus itself was in a node of a network connecting specific BAs and liver metabolism and inflammatory genes. Nevertheless, despite the above-stated starring role of *Akkermansia*, the microenvironment set up by the other changes observed in the gut microbiota of synbiotic-treated rats could also be behind the metabolic benefits observed.

This study presents a number of limitations. Firstly, regarding the analyzed samples, it could have been useful to collect not only white adipose tissue but also brown adipose tissue, due to its recently reported direct relationship with the beneficial effects of *A. muciniphila* [16]. Secondly, another limitation is directly related to the use of animal models, since some parameters associated with NAFLD development such as HOMA-IR have been developed in humans, being difficult to extrapolate to in vivo models. Thirdly, although we have observed an improvement in NAFLD related to quercetin supplementation, we have not deepened in its pharmacokinetic effects, as well as its metabolomic relationship with gut microbiota. Taking into account that several intestinal bacteria possess the capacity of quercetin biotransformation into low density derived polyphenols with antioxidant and anti-inflammatory properties [78,79], it could be interesting to include a metabolomic analysis which let us to elucidate this connection. Finally, although our results pointed out to a causal role of the modulation of gut microbiota in the improvement of early obesity and NAFLD, it could be interesting to go deeper into the specific contributing mechanistic relationship between lipid metabolism, gut-liver axis, gut microbiota, and bile acids signaling. Thus, additional studies are required to clarify the specific mechanisms that could be involved in the beneficial effects of this synbiotic.

Regarding future outlooks of our study, it could be useful to analyze the effects of another combinatory doses of the quercetin and *A. muciniphila* synbiotic, so this research could not only denote the most effective approach, but also possible toxic or unhealthy combinatory doses. Moreover, future experiments could analyze long-term effects of the synbiotic, in order to verify that the beneficial effects of the intervention were maintained over time. Furthermore, we have to emphasize that our intervention was performed in a juvenile model of obesity and NAFLD, and the beneficial observed effects could be different than those in an adult model, worthy to be studied. Finally, the effects of the combination of quercetin and *A. muciniphila* would be analyzed in patients in order to determine, not only its usefulness in the management of the disease, but also the security and efficacy of the proposed synbiotic approach.

## 5. Conclusions

Overall, the remarkable modulation of the BA profile, together with the upregulation of their synthesis and enterohepatic flux, as well as a modulation in hepatic inflammatory status and lipogenesis and fat storage process in white adipose tissue, could denote novel mechanisms behind the well-known beneficial effects of *A. muciniphila* in metabolic disease and support its use in combination with quercetin as a feasible strategy to face NAFLD and obesity development.

## Figures and Tables

**Table 1:** Effect of six weeks of HFD feeding on parameters related to early obesity and NAFLD development.

	C	HFD
Body weight (g)	418.36 ± 5.37	440.58 ± 6.85 *
Food intake (g/day)	20.42 ± 0.68	17.94 ± 0.81 ***
Food intake (kcal/day)	77.92 ± 1.30	93.08 ± 1.85 ***
ALT (U/L)	27.26 ± 1.78	48.28 ± 2.60 ***
AST (U/L)	114.19 ± 7.05	115.17 ± 4.46
Bilirubin (mg/dL)	1.09 ± 0.06	1.10 ± 0.09
Cholesterol (mg/dL)	56.76 ± 1.92	62.97 ± 2.28 *
LDL cholesterol (mg/dL)	2.58 ± 0.26	3.55 ± 0.27 *
Plasma albumin (g/L)	34.68 ± 0.49	34.76 ± 0.45
Fasting blood glucose (mg/dL)	122.48 ± 3.61	112.97 ± 2.49 *
Plasma insulin (ng/mL)	4.39 ± 0.33	6.86 ± 0.83 *
HOMA-IR	1.17 ± 0.12	2.02 ± 0.35 *

Values are represented as mean and standard error of the mean (SEM). * *p* < 0.05 vs. C; *** *p* < 0.001 vs. C. ALT, alanine aminotransferase; AST, aspartate aminotransferase; HOMA-IR, homeostatic model assessment for insulin resistance; LDL, low-density lipoprotein.

## Data Availability

Data is contained within the article.

## Supplementary Materials

The following are available online at https://www.mdpi.com/article/10.3390/antiox10122001/s1, Table S1: Primers used for RT-qPCR analysis, Table S2: Effect of 3 weeks of treatment with quercetin, *A. muciniphila* or synbiotic combination on parameters related to obesity and NAFLD development, Table S3: Pearson correlations between gut microbiota abundance, bile acids and liver gene expression, Figure S1: Gut microbiota composition after six weeks of HFD. Figure S2: Differences in fecal short chain fatty acids concentrations between before (6th week) and after the intervention (9th week), Figure S3: Associations of *Akkermansia* levels with plasma BAs and with hepatic gene expression.
