# Supplementary material for: The Synbiotic Combination of Akkermansia muciniphila and Quercetin Ameliorates Early Obesity and NAFLD through Gut Microbiota Reshaping and Bile Acid Metabolism Modulation
Source: Antioxidants (Basel). 2021 Dec 15;10(12):2001. doi: 10.3390/antiox10122001 (PMC8698339; doi:10.3390/antiox10122001)
Supplement: Supplementary file 1 [file antioxidants-10-02001-s001.zip › antioxidants-1472388-supplementary.pdf]

Supplementary information

The synbiotic combination of *Akkermansia muciniphila* and quercetin ameliorates early obesity and NAFLD through gut microbiota reshaping and bile acid metabolism modulation

María Juárez-Fernández[1], David Porras[1], Petar Petrov[2,3], Sara Román-Sagüillo[1], María Victoria García-Mediavilla[1,3], Polina Soluyanova[2], Susana Martínez-Flórez[1], Javier González-Gallego[1,3], Esther Nistal[1,3], Ramiro Jover[2,3,4‡] and Sonia Sánchez-Campos[1,3‡*]

[*]Shared senior authorship
[1]Institute of Biomedicine (IBIOMED), University of León, León, Spain.
[2]Experimental Hepatology Unit, IIS Hospital La Fe, Valencia, Spain.
[3]Centro de Investigación Biomédica en Red de Enfermedades Hepáticas y Digestivas (CIBERehd), Instituto de Salud Carlos III, Madrid, Spain.
[4]Biochemistry and Molecular Biology Department, University of Valencia, Valencia, Spain.

SUPPLEMENTARY INFORMATION

Table S1 **2**
Table S2 **3**
Table S3 **4**
Figure S1 **5**
Figure S2 **6**
Figure S3 **7**

Table S1. Primers used for RT-qPCR analysis.

Rat primers	Sense primer sequence (5´-3´)	Antisense primer sequence (5´-3´)
Baat	GATCCATGTAAAGGAAGGCCGAA	GGCCCGGAACTCAAACAGTC
Bsep	TGGGGCTCGTCAGATAAGGA	ACATGCGCTGGAGGAAATGA
Cebpa	TGGCTCTGGGTCTGGAAAGAA	AGAGAAGGAAGCAGTCCACCC
Cyp7a1	CAAGTGCAACTGAATGACCTGC	GAAGTCCTCCTTAGCTGTGCG
Cyp8b1	TCCCCAGGTTTGTCTACTCCC	CCAGGTTTTGCTCCACAGAGA
Dgat2	ATGGCCGAGGTCTCTTCTCC	CTCCAGCTTAGGGACGGTGA
Fatp5	TTTGACATAGAGACGGCAGAGC	GGTTTCATCCTGGGAACCACG
Frx	ACACGCAGACCTGTTGGAAG	TCATTTTCAGCTCCCCGACAC
Il1β	ACAGAACATAAGCCAACAAGTGGT	GTGGGTGTGCCGTCTTTCAT
Il6	CCCACCAGGAACGAAAGTCAA	TGGCTGGAAGTCTCTTGCG
Lxrα	GTGAGAGCATCACCTTCCTCAA	GAGCAGAGCAAATTCAGCATCAT<br>T
Mdr2	CAGCATCGCCAAGAACATCG	CAGAGAGCTGTGTCCCCTTG
Mrp2	ATTGGACTGCACGACCTTCG	TCCTCAGACTCCCCGAGAAC
Ntcp	TACTGGCTACCTCCTCCCTG	TTTCCATGCTGATGGTGCGT
Pxr	AATCTGCCGTGTATGTGGGG	CATTGCGTTTCATGGCCCTT
Scd1	CGGGAGAATATCCTGGTTTCCC	TCAGAAGCCCAAAGCTCAGCTAC
Shp	GGCACTATCCTCTTCAACCCA	TCCAGGACTTCACACAATGCC
Srebp1c	GTCAGTTCCAGCATGGCTACC	CACTTGGGGAATGTGCTCTACC
Srebp2	ACAGTTTCCGCCCAGCATAC	CTGGCTCCCGCCATTAGTC
Actb	AGGAGATTACTGCCCTGGCT	CAGCTCAGTAACAGTCCGCC
Pgk1	GCAGATTGTTTGGAACGGTCC	TAGTGATGCAGCCCCTAGACGT
Pparα	AGACTAGCAACAATCCGCCTTT	TGGCAGCAGTGGAAGAATCG
Ptges3	TTGGAAAGATTGGGAGGATGACTC	TCTGCTCCGTCTACTTCTGGT
Rplp0	ACAACCCAGCTCTGGAGAAA	TGCCCCTGGAGATTTTAGTG
Tlr2	AAGAGCATCGGCTGGAGGTC	GAGCTGCCATCACACACACC
**Bacterial primers**	**Sense primer sequence (5´-3´)**	**Antisense primer sequence (5´-3´)**
**16S rRNA926F-1062R (total bacteria)**	AAACTCAAAKGAATTGACGG	CTCACRRCACGAGCTGAC
**16S rRNA V3-V4**	TCGTCGGCAGCGTCAGATGTGTATAA<br>GAGACAGCCTACGGGNGGCWGCAG	GTCTCGTGGGCTCGGAGATGTGT<br>ATAAGAGACAGGACTACHVGGG<br>TATCTAATCC
***Akkermansia muciniphila* (AM1-AM2)**	CAGCACGTGAAGGTGGGGAC	CCTTGCGGTTGGCTTCAGAT

Abbreviations: *Baat*, Bile acid-CoA:amino acid N-acyltransferase; *Bsep*, Bile salt export pump; *Cebpa*, CCAAT/enhancer-binding protein alpha; *Cyp7a1*, Cytochrome P450 family 7 subfamily A member 1; *Cyp8b1*, Cytochrome P450, family 8, subfamily b, polypeptide 1; *Dgat2*, Diacylglycerol O-acyltransferase 2; *Fatp5*, Fatty acid transport protein 5; *Il1β*, Interleukin 1 beta; *Il6*, Interleukin 6; *Fxr*, Farnesoid X receptor; *Lxrα*, Liver X receptor alpha; *Mdr2*, Multidrug resistance protein 2; *Mrp2*, Multidrug resistance-associated protein 2; *Ntcp*, Na^+/taurocholate cotransporting polypeptide; *Pxr*, Pregnane X receptor; *Scd1*, Stearoyl-CoA desaturase-1; *Shp*, Small heterodimer partner; *Srebp1c*, Sterol regulatory element-binding protein 1c; *Srebp2*, Sterol regulatory element-binding protein 2; *Actb*, Actine beta; *Pgk1*, Phosphoglycerate kinase 1; *Pparα*, Peroxisome proliferator-activated receptor Alpha; *Ptges3*, Prostaglandin E synthase 3; *Rplp0*, Ribosomal Protein Lateral Stalk Subunit P0; *Tlr2*, Toll-like receptor 2.

Table S2. Effect of 3 weeks of treatment with quercetin, *A. muciniphila* or synbiotic combination on parameters related to obesity and NAFLD development.

	**C**	**C[HFD]**	**C[HFD]+Q**	**C[HFD]+A**	**C[HFD]+Q+A**
**Body weight** (g)	505.65 ± 6.81	491.85 ± 10.68	501.14 ± 14.04	536.38 ± 16.90	493.88 ± 20.23
**Food intake** (g/day)	20.99 ± 0.43	20.57 ± 0.57	21.84 ± 0.30	22.95 ± 1.28	20.86 ± 0.77
**Food intake** (kcal/day)	80.21 ± 1.63	78.57 ± 2.17	83.43 ± 1.14	87.67 ± 4.88	79.70 ± 2.92
**ALT** (U/l)	31.53 ± 1.40	34.66 ± 2.38	36.93 ± 1.99	35.50 ± 2.53	39.47 ± 4.92
**AST** (U/l)	99.75 ± 13.12	106.10 ± 24.91	78.84 ± 8.20	97.25 ± 10.40	98.77 ± 3.87
**Cholesterol** (mg/dl)	63.79 ± 3.11	66.47 ± 11.89	75.78 ± 6.10	70.81 ± 3.36	70.25 ± 5.81
**Plasma albumin** (g/l)	26.86 ± 0.29	25.48 ± 1.32	26.30 ± 0.64	25.25 ± 0.41*	28.27 ± 1.27
**Fasting blood glucose** (mg/dl)	135.89 ± 2.50	152.17 ± 5.78*	151.29 ± 7.09*	134.75 ± 4.61#	130.25 ± 6.06#
**Plasma insulin** (ng/ml)	11.12 ± 0.93	15.91 ± 0.62*	14.09 ± 1.06	13.86 ± 1.46	12.78 ± 1.73
**HOMA - IR**	4.11 ± 0.29	5.91 ± 0.34*	5.30 ± 0.36	4.51 ± 0.45	3.58 ± 0.50#

Values are represented as mean and standard error of the mean (SEM). *$p < 0.05$ *vs*. C; #$p < 0.05$ *vs*. C[HFD]. ALT, alanine aminotransferase; AST, aspartate aminotransferase; HOMA-IR, homeostatic model assessment for insulin resistance.

Table S3. Pearson correlations between gut microbiota abundance, bile acids and liver gene expression.

r Pearson	*Actinobacteria*	*Cyanobacteria*	*Bacilli*	*Blautia*	*Coprobacillus*	*Lactibacillus*	*Lactococcus*	*Oscillospira*	*Roseburia*	CEBPA	SREBP1c	SREBP2	DGAT2	SCD1	LXRα	FXR	NTCP	BSEP	MDR2	MRP2	CYP8B1	FATP5	BAAT
**CA**									**-0.37**														
**αMCA**									-0.42	-0.32													
**βMCA**		0.36		-0.33			-0.30		-0.37	**-0.55**		**-0.42**	-0.36	0.35	0.31			0.38			**0.60**	**0.44**	0.38
**DCA**				0.33	**0.64**					0.35													
**HDCA**									**0.46**	**0.49**								0.33				-0.36	
**ωMCA**							-0.38									**0.54**	**0.61**	**0.57**	**0.56**	**0.34**	**0.71**	**0.57**	**0.59**
**Total BAs**										-0.22	**0.48**												
**Primary BAs**									-0.46	**-0.40**												0.33	
**CA/DCA**		**0.44**								**-0.41**													
**CA/(GCA +TCA)**																0.33	**0.65**	**0.60**	0.37	0.36	**0.65**	0.33	**0.53**
Il-6					**-0.66**				**0.48**														
Tlr2	**0.45**																						
Pparα					**0.66**																		
Cebpa	0.40	**-0.45**							0.35														
Srebp1c			-0.36			-0.37	**-0.42**	0.35															
Srebp2	**0.43**																						
Dgat2	0.38							0.40															
Scd1	-0.37	**0.55**																					
Lxrα					**-0.40**																		
Ntcp									-0.32														
Bsep		0.41																					
Cyp7a1		0.33																					
Cyp8b1		0.33							-0.35														
Baat		0.31																					

Correlation coefficients in bold are statistically significant (p<0.05)

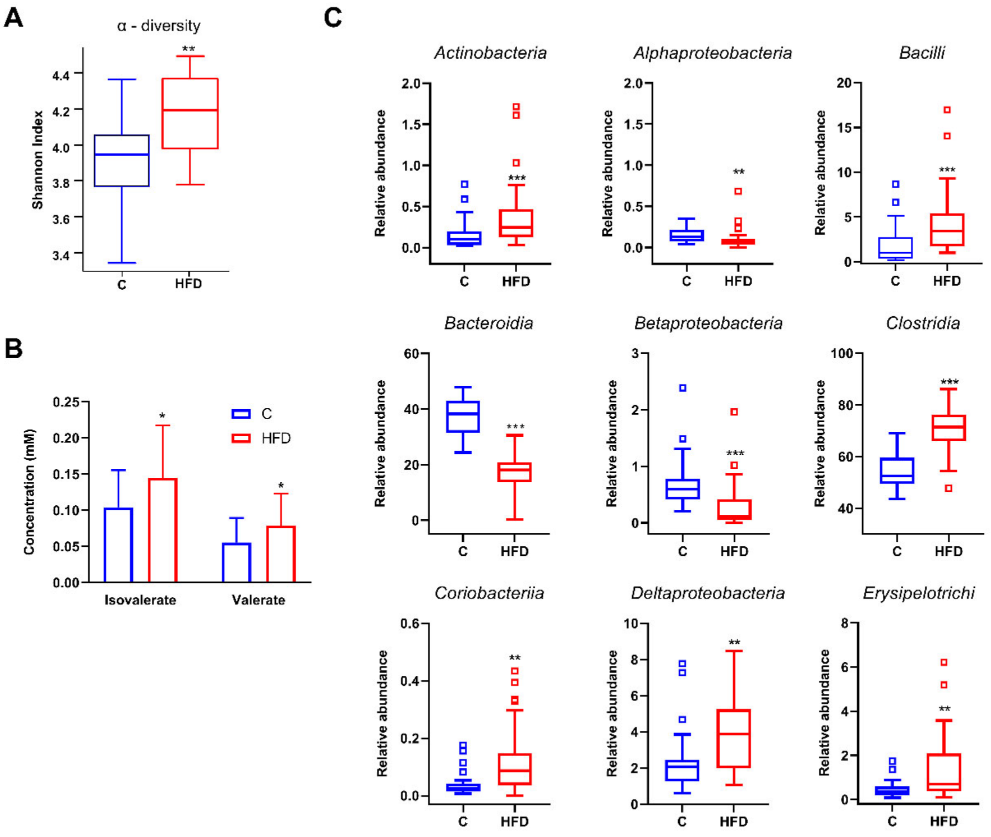

Figure S1. (A) Box plot of alpha diversity measured by Shannon index at 6th week. **(B)** Bar graphs showing the differences related to valeric and isovaleric fecal concentrations between control and HFD-fed rats. **(C)** Box plot showing differences in the relative abundance at class level between C and HFD groups at 6th week. Statistical analysis was performed using Kruskal-Wallis followed by Mann-Whitney U test (p<0.05). $^{*}p$<0.05, $^{**}p$<0.01 *vs* C; $^{\#}p$<0.05 *vs* HFD.

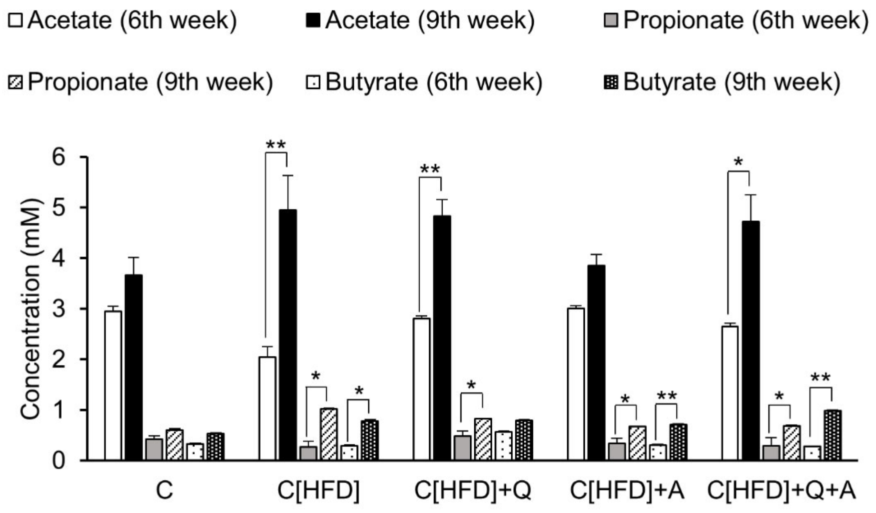

Figure S2. Differences in fecal short chain fatty acids concentrations between before (6th week) and after the intervention (9th week). Statistical analysis was performed using paired sample t test or non-parametric Wilcoxon signed-rank test. *$p<0.05$, **$p<0.01$ (9th *vs* 6th week).

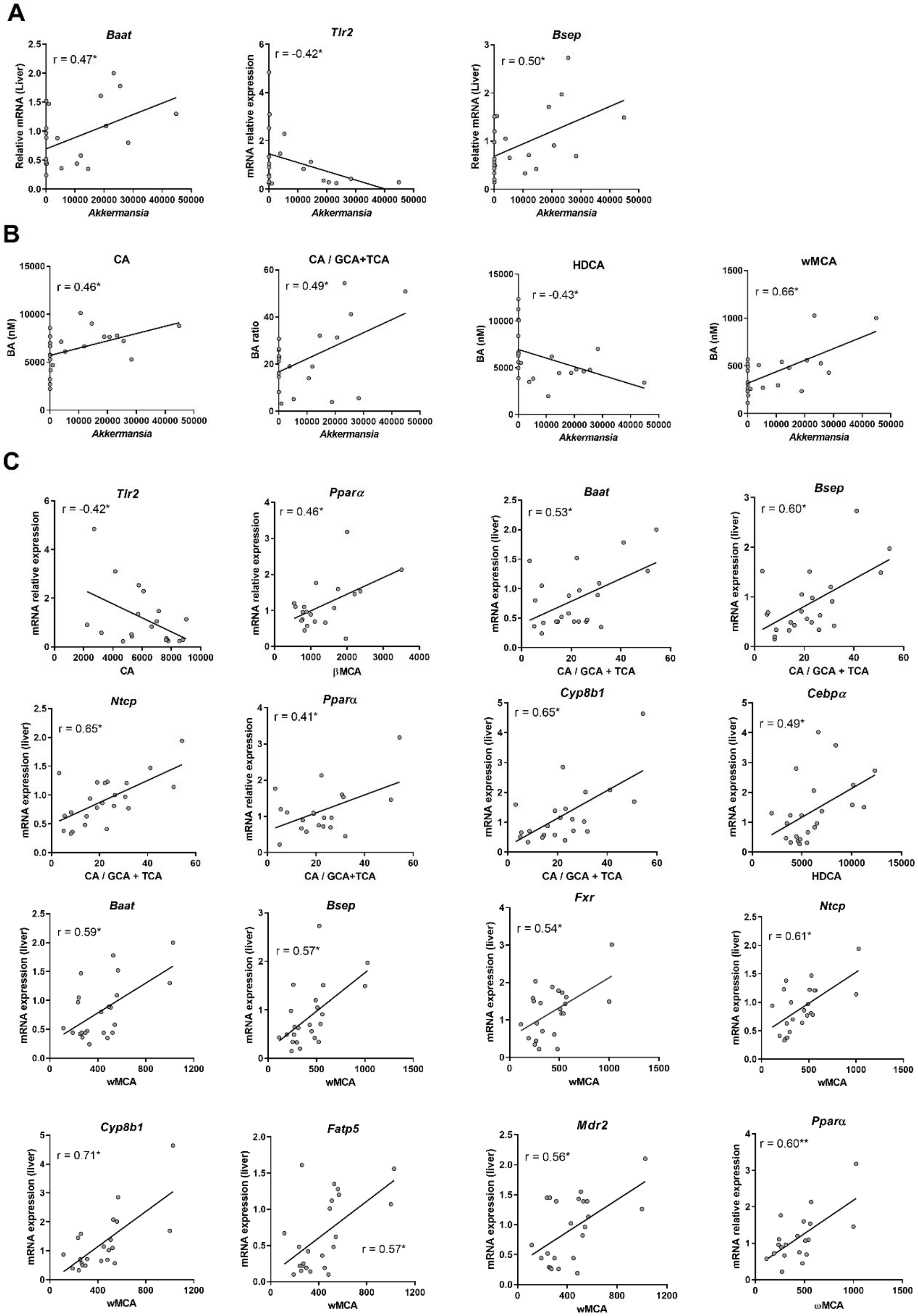

Figure S3. Associations of *Akkermansia* levels with plasma BAs and with hepatic gene expression. Correlation coefficients and linear relationships of **(A)** *Akkermansia* genus gut normalized reads with hepatic mRNA levels; **(B)** *Akkermansia* genus gut normalized reads with plasma BAs and BA ratios; **(C)** plasma BAs and BA rations with mRNA levels of hepatic genes; $^{*}p < 0.05$.
